# Network-based analysis of diagnosis progression patterns using claims data

**DOI:** 10.1038/s41598-017-15647-4

**Published:** 2017-11-14

**Authors:** Eugene Jeong, Kyungmin Ko, Seungbin Oh, Hyun Wook Han

**Affiliations:** 1 Ajou University School of Medicine, Department of Biomedical Informatics, Suwon, 16499 Republic of Korea; 2 Medstar Georgetown University Hospital, Department of Pathology, Washington, DC 20007 USA

## Abstract

In recent years, several network models have been introduced to elucidate the relationships between diseases. However, important risk factors that contribute to many human diseases, such as age, gender and prior diagnoses, have not been considered in most networks. Here, we construct a diagnosis progression network of human diseases using large-scale claims data and analyze the associations between diagnoses. Our network is a scale-free network, which means that a small number of diagnoses share a large number of links, while most diagnoses show limited associations. Moreover, we provide strong evidence that gender, age and disease class are major factors in determining the structure of the disease network. Practically, our network represents a methodology not only for identifying new connectivity that is not found in genome-based disease networks but also for estimating directionality, strength, and progression time to transition between diseases considering gender, age and incidence. Thus, our network provides a guide for investigators for future research and contributes to achieving precision medicine.

## Introduction

The last few decades have witnessed notable advances in our understanding of human diseases. The volume and complexity of biological and clinical data have grown extensively, and many researchers have sought to investigate the risk factors and root causes of diseases using various approaches^1–4^. Multiple causes and risk factors of diseases have been defined, and the evidence for relationships between diseases has been summarized in several dimensions. Among various methods, network analysis has been considered an effective strategy for summarizing the relationships among diseases^1–9^. Disease networks based on genetic and proteomic databases and disease phenotypic networks using comorbidity information from claims data have been introduced. For instance, Goh *et al*. constructed a human disease network (HDN) using disease associations on the basis of shared genes^3^, and Hidalgo *et al*. built a phenotypic disease network (PDN) based on a set of comorbidity associations between human diseases using Medicare claims data^4^. These networks have served as guidelines for many other studies and are suggestive of a disease-disease connectivity map.

Although progress in disease network construction has been notable, challenges remain in bridging the gap between network research based on biological databases and clinical practice: (I) Genetic disease networks in which links represent shared genes or protein-protein interactions (PPIs) cannot explain relationships in non-genetic disorders, such as bone fracture. (II) Significant risk factors, such as the order of incidence, gender and age, must be considered when investigating the relationships between diseases^10–14^. Additionally, genetic disease networks cannot provide detailed explanations of the weights and directionality of disease-disease associations, sex-specific diseases, and age-dependent diseases since biological data are not sequential or specific to gender and age. Furthermore, (III) the durations of disease-disease relationships cannot be defined.

Claims data include the diagnosis history of both genetic and non-genetic diseases and information on risk factors such as sex and age. In addition, the duration of progression can be identified because the data provide chronological medical histories of patients. Therefore, we established a diagnosis progression network to inform the relationships between diseases using the National Health Insurance Service of Korea (NHIS) sample cohort data (see Methods).

## Results

### Construction of the diagnosis progression network (DPN)

To identify the patterns of diagnosis progression, the DPN was visualized using Cytoscape to show complex relationships and processes in a weighted and directed graph. In the DPN, nodes represent diagnoses identified by International Statistical Classification of Diseases and Related Health Problems 10th Revision (ICD-10) codes, which constitute the diagnostic classification standard. Under the assumption that a prior diagnosis would serve as a risk factor for a later diagnosis, we measured the strengths of relationships between nodes by introducing the notion of “relative risk” (RR), which is the ratio of the probability of an event occurring in one group compared to the probability of an event occurring in another group, and diagnoses were connected if they showed significant temporal relationships under Bonferroni-corrected Fisher’s exact test (see Methods). Since the DPN was constructed considering directionality, the degree of a node was determined by its in-degree (the number of incoming edges from other nodes) and out-degree (edges emanating from the node). Meanwhile, the relationship between two nodes was classified into two groups: unidirectional and bidirectional. We further divided bidirectional relationships into another two categories based on the ratio of the RRs (or weights) in each direction.

**Figure 1:**
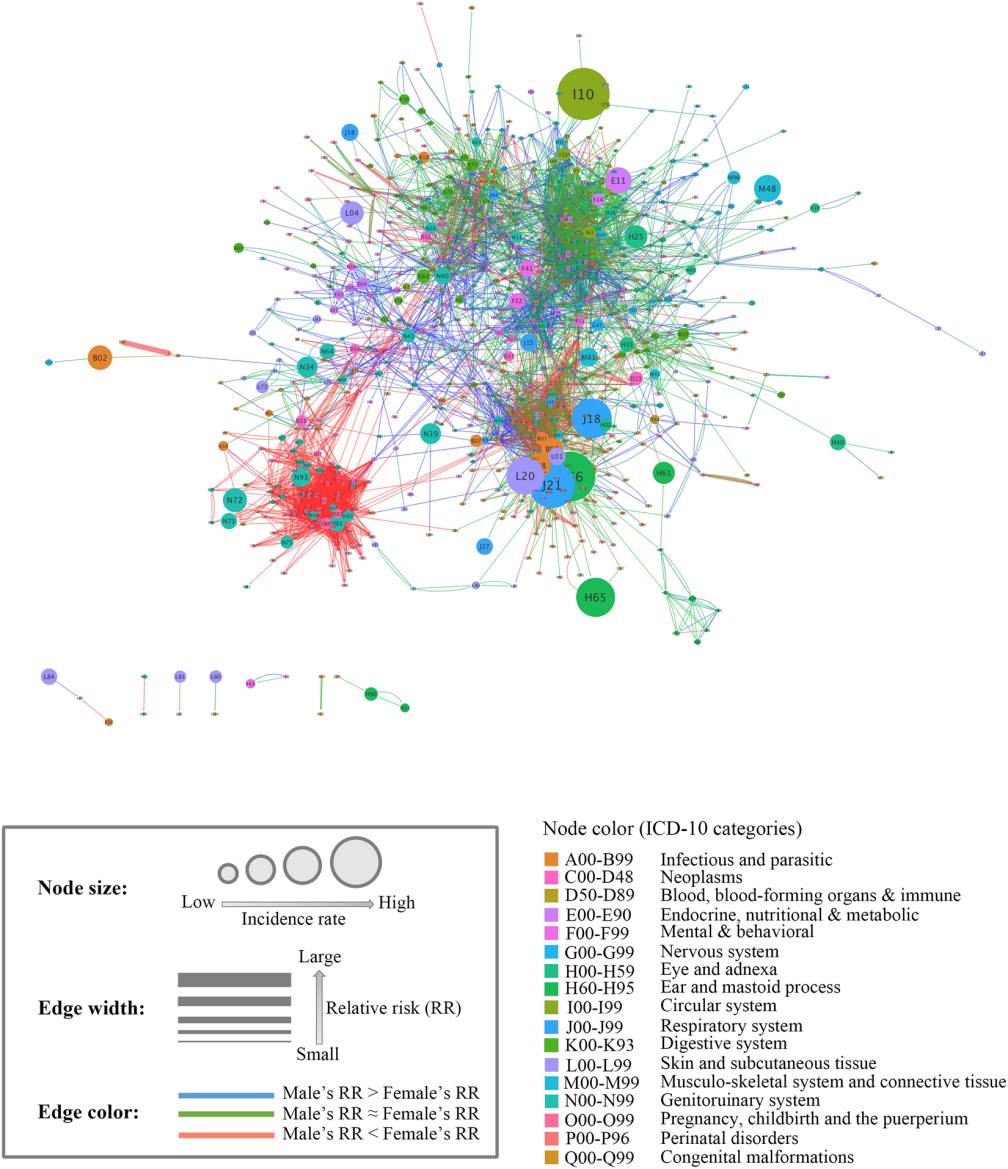
The DPN. Node size is proportional to disease incidence, and the color of the nodes corresponds to the ICD-10 disease category. The width of an edge is proportional to the size of the RR, and the color of an edge indicates the difference between the female RR and the male RR: If the female RR is two-fold greater or more than the male RR, the links are red. If the male RR is two-fold greater or more than the female RR, the links are blue. If the RRs are similar, the links are green.

If the RRs of the two edges of a bidirectional relationship differed by twofold or greater, the pair was classified as a “lop-sided” bidirectional relationship. Otherwise, bidirectional relationships were classified as “even”. The directionalities and weights (RRs) of statistically significant node pairs are given in Supplementary Table S1. To investigate whether the RR of each edge differed by gender, the RRs of all edges in the DPN were calculated by gender after stratifying the NHIS cohort data by sex. If the RR of females was at least two times greater than that of males, then the edges were female-dominant (red). If the RR of males was at least twice as large as that of females, then the edges were male-dominant (blue). If they were similar, the links were not sex-dominant (green). The 12-year average incidence of each diagnosis was used as an attribute of the node. The resulting DPN included 775 diagnoses (17 ICD-10 categories) and 4,100 relationships between diagnosis pairs (2,464 unidirectional relationships, 1,335 even bidirectional relationships, and 301 lop-sided bidirectional relationships) formed by 5,736 edges (1,868 female-dominant edges, 329 male-dominant edges, and 3,539 edges that were not sex-dominant). The incidences of diagnoses in the network ranged from 3 to 17,736 per year, and the RRs of the edges ranged from 4 to 2,854.27 (Fig. 1).

An illustration of the network is shown in Fig. 1. Although we did not manually modify or locate the nodes in the network, it is clear that groups of nodes clustered together under the prefuse force-directed layout. Except for the pregnancy-related diseases category, there was no tendency of clustering by ICD-10 category; instead, the ICD-10 categories were distributed throughout the network.

### Network characterization

To explore and understand the properties of the DPN, it is necessary to answer the following three questions: Q1) What are the topological characteristics of our network? Q2) Which factors determine the structure and function of the DPN? Q3) How does the DPN differ from existing networks? First, we endeavored to answer the first question (Q1) by analyzing the in-, out-, and combined-degree distributions, which are the most fundamental topological properties of a network. To answer the second question (Q2), we investigated the influences of age, gender, and ICD-10 categories on the network using community detection and by examining how long it takes for one diagnosis to progress to another. Lastly, we explored the third question (Q3) by constructing a genome-based disease network (GBDN) using shared genes and PPIs to investigate the differences between the DPN and the GBDN.

**Figure 2:**
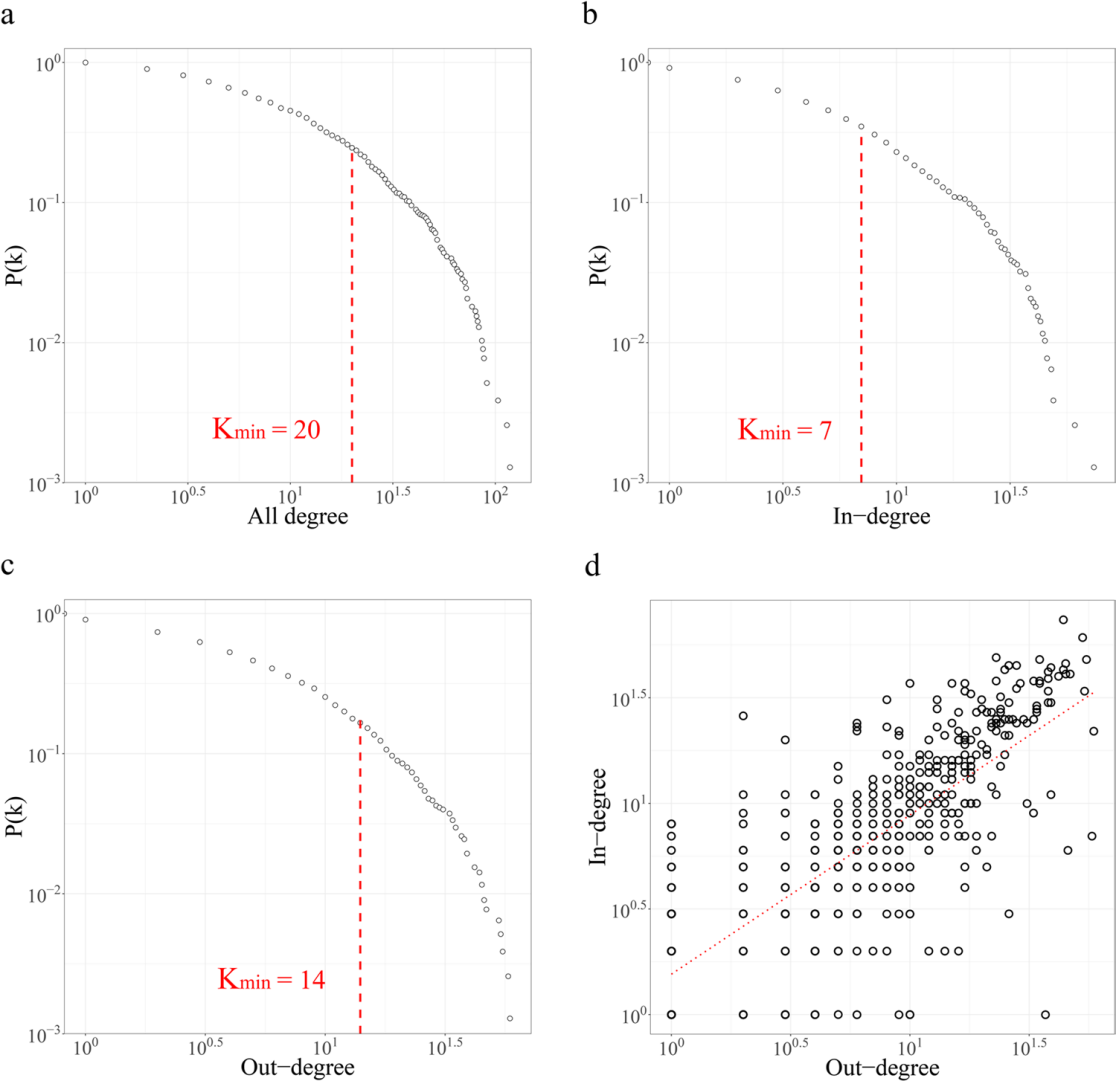
The properties of degree distribution. (**a**) The cumulative degree distribution of the DPN with exponent **γ** = **2**.**65** (**b**) The cumulative in-degree distribution of the DPN with exponent **γ**_***in***_ = **2**.**32**. (**c**) The cumulative out-degree distribution of the DPN with exponent **γ**_***out***_ = **2**.**95**. (**d**) The positive correlation between the in- and out-degrees of diagnoses.

We examined the topological characteristics of our network considering the degrees of the nodes and the weights and the disease-disease duration of the edges. We examined the distributions of connectivity for each case where directionality is considered (in/out-degree) and where it is not (combined degree). Our results show that the DPN is a typical scale-free network with a combined degree distribution that follows a power law (${{\rm{\gamma }}}_{{all}}=2.65$, ${x}_{{\min }}=20$, $p=0.24$), indicating that a few diseases such as chronic kidney disease (*k*_*all*_ = 127) and heart failure (*k*_*all*_ = 86) are highly connected to other diseases, whereas most diseases share relatively few links (Fig. 2a). The high *k*_*all*_ of these diagnoses are explained by the fact that both heart failure and chronic kidney disease are well known to elevate the risk of other complications, and comorbidity is common in patients with CHK or HF^15–20^. Additionally, most of the top-ranking high-degree diagnoses were complex diseases caused by the interaction of multiple factors, explaining the small number of high-degree diagnoses. Meanwhile, the in- and out-degree distributions of nodes also followed power laws (γ_*in*_ = 2.32, *x*_*min*_ = 7, *p*_*in*_ = 0.19, γ_*out*_=2.95, *x*_*min*_ = 14, *p*_*out*_ = 0.42), similarly showing that there were a few high in-degree or high out-degree diagnoses (Fig. 2b,c). The top 10 k_all_, k_out_ and k_in_ diagnoses are listed in Supplementary Table S2.

To investigate the relationship between the degrees considering directionality, we drew a scatter plot of the in-degrees and out-degrees of nodes and drew a simple linear regression line. A Spearman correlation analysis was performed to statistically analyze the correlation between the two variables. Figure 2d shows that a strong positive correlation existed between these variables (adjusted r = 0.799 *p* = 4.436e^−21^), illustrating that the diagnoses that lead to many other diagnoses tend to have many incoming edges. As a representative example, “Dementia in Alzheimer’s disease” (F00) and “Sequelae of cerebrovascular disease” (I69) both showed high numbers of the outgoing edges (*k*_out_ = 57 and *k*_out_ = 43) and incoming edges (*k*_in_ = 48 and *k*_in_ = 43). In contrast, “Subclinical iodine-deficiency hypothyroidism” (E02) and “Optic neuritis” (H46) were found to have low in-degree and low out-degree nodes (*k*_out_ = 3 and *k*_out_ = 3; *k*_in_ = 2 and *k*_in_ = 4). However, there were some diagnoses with in-degrees and out-degrees that were extremely different. For example, “Cardiac arrest” (I46), “Respiratory failure” (J96), and “Secondary malignant neoplasm of other sites” (C79), which are well known to have high mortality rates, showed relatively high in-degree nodes (*k*_in_ = 26, *k*_in_ = 20, *k*_in_ = 22, respectively) compared to out-degree nodes (*k*_out_ = 2, *k*_out_ = 3, *k*_out_ = 6, respectively), whereas menstrual-related diagnoses, such as “Excessive, frequent and irregular menstruation” (N92) and “Pain and other conditions associated with female genital organs and the menstrual cycle” (N94), which have unknown fundamental causes, showed high out-degree nodes (*k*_out_ = 46, *k*_out_ = 37, respectively) and very low in-degree nodes (*k*_in_=6, *k*_in_=1, respectively).

**Figure 3:**
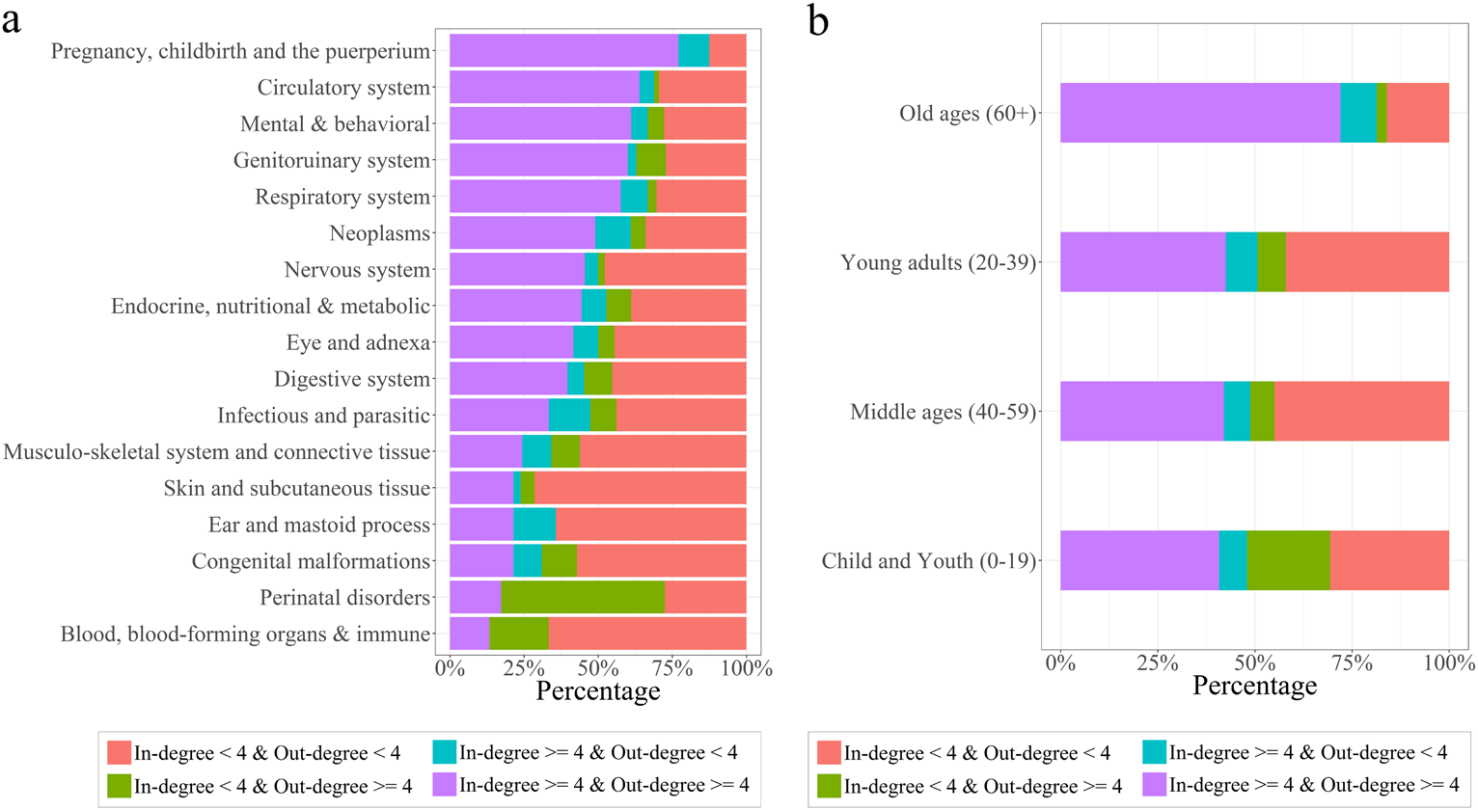
Degree distribution analysis by ICD-10 categories and age groups. (**a**) The in- and out-degree distribution analysis across 17 ICD-10 categories. (**b**) The in- and out-degree distribution analysis across 4 age groups.

For a more global analysis of which factors were involved in the degree distributions, we initially grouped the diagnoses into four “degree” groups based on the sizes of the in-degree and the out-degree relative to the median values: the high in-degree (≥4) and high out-degree (≥4) group, the high in-degree (≥4) and low out-degree (<4) group, the low in-degree (<4) and high out-degree (≥4) group, and the low in-degree (<4) and low out-degree (<4) group. Finally, we compared the proportions of degree groups in each ICD-10 category and age group. The results showed that the proportions differed for each ICD-10 category (Fig. 3a). We conducted an enrichment analysis to determine whether the four degree groups were significantly enriched in each ICD-10 group or in each age group. The “Pregnancy, childbirth and puerperium category” (O00-O99) showed the largest proportion of diagnoses with both high in-degree and high out-degree nodes (*p*-value = 1.22e-09), while more than 70% of nodes in the “Skin and subcutaneous tissue disorders” (L00-L99) category showed both low in-degree and low out-degree nodes (*p*-value = 1.47e-07). The low in-degree and high out-degree group was significantly enriched in only “Certain conditions originating in the perinatal period” (P00-P96) (*p*-value = 1.148e-10). In addition, there were no high in-degree and low out-degree nodes in this category. Notably, the proportions of the four degree groups also differed for each age group. The old-age (60 + ) group was the only group with enriched high in-degree and high out-degree diagnoses and showed the largest proportion of high in-degree and high out-degree nodes (*p*-value = 4.46e-11, Fig. 3b). Low in-degree and high out-degree diagnoses were enriched in the childhood and adolescence (0–19) age group (*p*-value = 1.62e-03). Meanwhile, low in-degree and low out-degree diagnoses were enriched in the young adult (20–39) group and in the middle-age (40–59) group (*p*-value = 1.125e-02, *p*-value = 2.54e-07, respectively).

### Clustering analysis of the DPN

**Figure 4:**
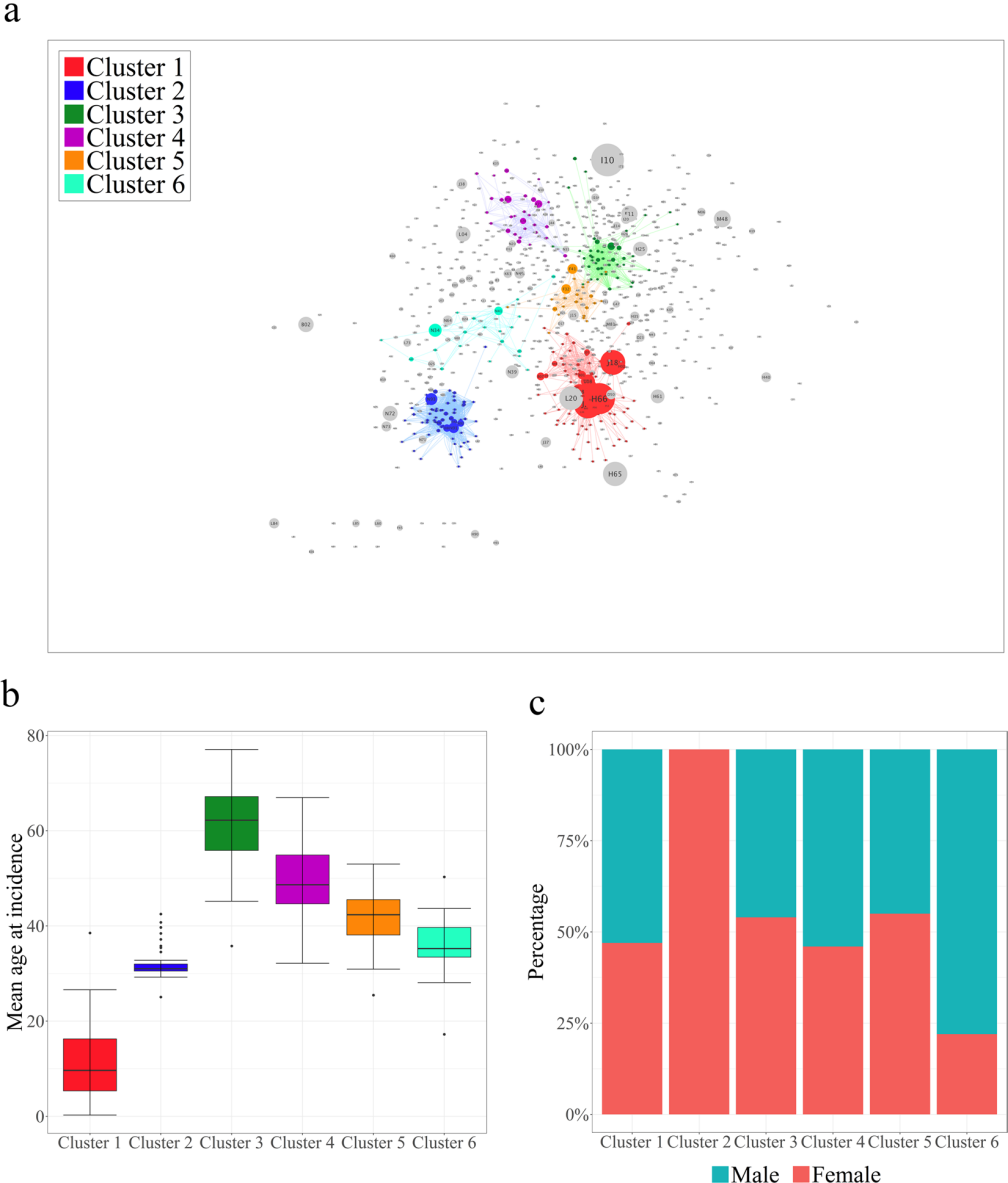
The results of community detection in the DPN using the Infomap algorithm. (**a**) Graphical view of 6 giant clusters detected by the Infomap algorithm. (**b**) Boxplot of the mean age at incidence for each of the 6 giant clusters. (**c**) Gender distribution in each of the 6 giant clusters.

Community detection is one of the key steps in investigating network properties in depth. A network is said to have community structure if the nodes are naturally divided into densely interconnected subgroups that are sparsely connected to each other. To determine the clustering characteristics of the DPN, we used the Infomap method in the *igraph* package to detect highly interconnected regions in our directed and weighted network (see Methods). Since the Infomap method is based on the random walk theory, we selected the highest modularity partition from 100 runs of the Infomap algorithm. Modularity, which is a measurement of the strength of division of a network into clusters, was 0.79, suggesting that the DPN has certain divisions (see Methods). Of 775 nodes, 444 nodes formed 88 clusters, including six giant clusters (over 20 nodes) that covered 77, 64, 44, 28, 23 and 21 diagnoses (Fig. 4a).

We found that each giant cluster had distinct properties. For instance, cluster 2 was composed entirely of female-specific diagnoses, such as those in the “Pregnancy, childbirth and puerperium” (O00-O99) category (0 male patients, Fig. 4c), whereas cluster 6 was composed of mostly male-specific diseases related to the genitourinary system (the ratio of females to males was 1:1.82, Fig. 4c). Meanwhile, cluster 1 and cluster 3 featured age-related characteristics: cluster 1 contained several childhood diseases such as “Other disturbances of temperature regulation of newborn” (P81) and “Varicella [chickenpox]” (B01), and the mean age of onset in this cluster was less than 12 years (mean age = 11.2 years, sd = 7.63, Fig. 4b), while cluster 3 contained chronic diseases that are common in old age, such as “Parkinson’s disease” (G20) and “Vascular dementia” (F01) (mean age = 61.5 years, sd = 8.00, Fig. 4b).

We performed an enrichment analysis using the Fisher exact test to identify which categories of diagnoses were over- or under-represented in each giant cluster. Except for cluster 1 and cluster 6, each cluster was enriched with distinct ICD-10 categories (Table 1). However, even though both cluster 1 and cluster 6 shared the infectious and parasitic category, the features of the infectious and parasitic diagnoses within each cluster were significantly different with respect to age. Cluster 1 was predominantly composed of infectious and parasitic diagnoses that usually occur before the age of 15 years, while cluster 6 mainly consisted of diagnoses related to old age. Cluster 5 was entirely composed of mental illness-related diseases. These results imply that our network was naturally clustered not only according to the specific ICD-10 categories but also according to age and gender.

**Table 1:** ICD-10 category enrichment analysis for each giant cluster.

Giant clusters	ICD-10 categories	*p*-value
Cluster 1	Perinatal disorders (P00-P96)	7.91e-15
	Infectious and parasitic (A00-B99)	5.72e-04
Cluster 2	Pregnancy, childbirth and the puerperium (O00-O99)	1.61e-59
Cluster 3	Circulatory system (I00-I99)	1.55e-03
	Nervous system (G00-G99)	4.04e-11
Cluster 4	Digestive system (K00-K93)	1.26e-09
Cluster 5	Mental and behavioral (F00-F99)	3.39e-29
Cluster 6	Infectious and parasitic (A00-B99)	0.00032
	Genitourinary system (N00-N99)	0.00016

### Duration to transition between diagnoses

Because the NHIS database includes chronological medical histories, we collected information on the length of time for each diagnosis-diagnosis edge in the DPN. The median diagnosis-diagnosis duration ranged from 0 to 2,617 days (7.27 years). Diagnosis progressions with the shortest median durations generally represent progressions to similar diagnoses, such as the progression from “Other sepsis” (A41) to “Bacterial sepsis of newborn” (P36), the progression from “Other appendicitis” (K36) to “Acute appendicitis” (K35), and the progression from “Intracerebral hemorrhage” (I61) to “Subarachnoid hemorrhage” (I60), likely due to the establishment of a definite diagnosis after a presumptive diagnosis. By contrast, the longest median intervals were mostly found between diseases that were not previously known to be associated. For example, the progression from “Spinal osteochondrosis” (M42) to “Unspecified dementia” (F03) was one of the longest duration pairs, and there was no study confirming a statistically significant correlation between them. Although the association between epilepsy and mental retardation is well known, few studies have addressed how long it will take epilepsy to progress to mental retardation. Five pairs with the top 10 longest median durations had “Disorders of puberty, NEC” (E30) as a later diagnosis, indicating that some diseases diagnosed in childhood lead to puberty-related diseases later in life. The distribution of durations differed markedly across age groups. The proportion of short-term progressions decreased steadily with age, while the proportion of medium-term progressions increased steadily. The long-term ratio remained almost constant. In addition, there were no differences between males and females, and medium-term progressions were the most common (Table 2). Detailed information on the durations of all progressions are listed in Supplementary Table S1.

**Table 2:** Distribution of pairs by age group and gender.

	Number of pairs according to age group				Number of pairs according to gender	
	Child and youth (0–19) (%)	Young adult (20–39) (%)	Middle age (40–59) (%)	Old age (60 + ) (%)	Male (%)	Female (%)
Short-term (<1 year)	1,327 (49.7)	1,890 (49.4)	1,835 (44.9)	1,217 (35.7)	1,687 (38.9)	2,147 (39.0)
Medium-term (1 − <3 years)	1,094 (41.1)	1,613 (42.1)	1,890 (46.2)	1,900 (55.7)	2,301 (53.0)	2,925 (53.1)
Long-term (3 + years)	240 (9.2)	325 (8.5)	364 (8.9)	293 (8.6)	351 (8.1)	433 (7.9)
Total	2,661	3,828	4,089	3,410	4,339	5,505

### Comparison between the DPN and the gene-disease network

Next, we compared the DPN and the gene-disease network to determine the correlation between the diagnosis-diagnosis connections and their shared genes or PPIs and to show that our network can suggest new interpretations of the relationships between diseases that the gene-disease network cannot. To construct the gene-disease network, we primarily integrated two databases, the Online Mendelian Inheritance in Man (OMIM) database and the STRING protein-protein interaction database (interaction confidence score of 0.8 or greater), similar to the gene-disease network constructed by Zhou *et al*.^21^. We connected nodes if they shared at least one gene or a PPI between diseases. The weights of edges were defined as the sum of the numbers of shared genes and PPIs.

It is important to note that OMIM disease names differ from ICD-10 diagnoses. Therefore, it is critical to map the OMIM disease names to the ICD-10 codes before comparison. We mapped OMIM disease names to ICD-9-CM codes based on the mapping table constructed by Park *et al*.^22^. ICD-9-CM codes were then mapped to ICD-10-CM codes, and finally ICD-10-CM codes were mapped to ICD-10 codes (Supplementary Table S3, see Methods). Moreover, to compare the differences in connectivity between the DPN and the gene-disease network, we selected only 29 cancer diseases. The cancer subgraph of the DPN consisted of 27 diagnosis pairs (14 even bidirectional pairs and 13 unidirectional pairs), while the cluster from the gene-disease network consisted of 261 pairs, corresponding to more than 64% of all possible combinations of the 29 nodes, indicating that the nodes of the gene-disease network were more tightly interconnected with each other.

Since the DPN is a directed network and the gene-disease network is not, it is impossible to compare links on a one-to-one basis unless the directionality of the edges is neglected. For this reason, we first converted the cancer subgraph of the DPN to an undirected graph, and then the average of the two RRs in the bidirectional edge was designated the RR of the new undirected edge. We evaluated the number of edges in the DPN subgraph that overlapped with those of the genes/PPIs network and found that 14 of 27 links in the DPN (51.9%) overlapped with the links in the genes/PPI network.

We further analyzed non-overlapping links and found that some of them were clinically meaningful. For example, for the link between “Colon cancer” (C18) and “Prostate cancer” (C61), even though there is clear evidence suggesting that patients with prostate cancer are more likely to develop colon cancer^23,24^, there was no connectivity in the gene-disease network. For the link between “Bladder cancer” (C67) and “Lung cancer” (C34), although there are many studies showing that patients with bladder cancer are more likely to have lung cancer^25–27^, connectivity was only evident in the DPN cancer subgraph.

To determine whether the RR and the number of shared genes and PPIs of each link were positively correlated, each pair of the DPN cancer subgraph was analyzed via Spearman correlation analysis, and the results showed that there were no certain correlations between them. For example, even though no gene or PPI was shared between “Cervical cancer” (C53) and “Uterine cancer” (C55), the RR of this pair was one of the highest in our network cluster (RR = 265.49). As another example, the pair of diagnoses “Stomach cancer” (C16) and “Liver and intrahepatic bile duct cancer” (C22) showed a moderately low RR close to the cutoff value (RR = 5.40), but the sum of the numbers of shared genes and PPIs was 25.

### Practical usability of the DPN

To facilitate the understanding of the analyses, the results were combined as edge attributes of the network. The data provided for each disease progression included four types of information: the distribution of cases across age groups; the distribution of cases by gender; duration distribution, including the median, maximum, minimum and standard deviation (sd) of the duration; and the RR, including the total RR and the gender-specific RR.

**Figure 5:**
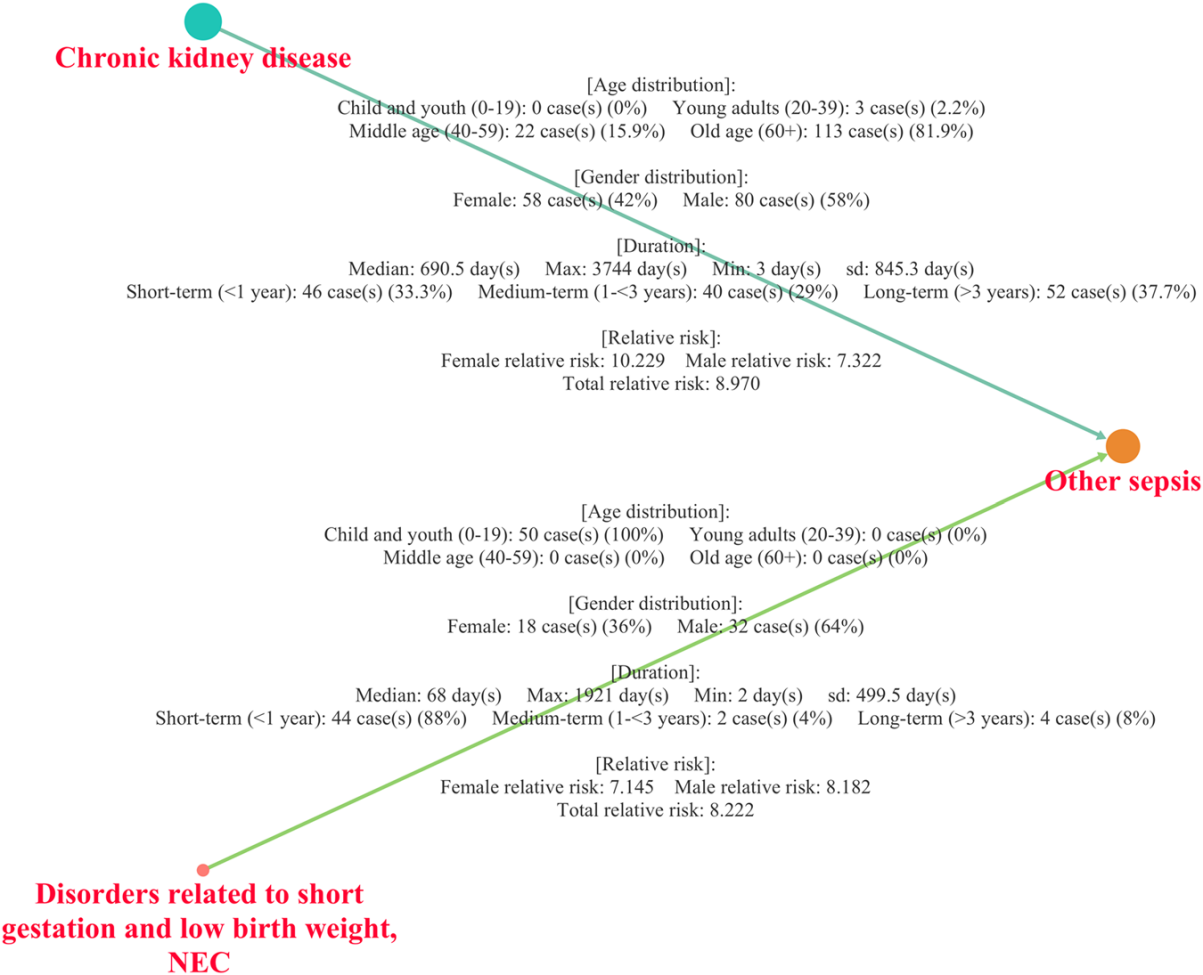
Two different progressions with the case distributions of age groups and gender and duration and RR information. Even though the two different progressions share an end node, which is “Other sepsis” (A41), they have different clinical information.

Although different prior diagnoses can lead to the same end diagnosis, the clinical information of each progression can vary considerably. For example, “Chronic kidney disease” (G03) and “Disorders related to short gestation and low birth weight” (J96) share “other sepsis” (A41) as a later diagnosis (Fig. 5). However, their durations, age distributions, and RRs in men and woman were notably different from each other. For instance, in the progression of “Disorders related to short gestation and low birth weight, NEC” (J96) to “Other sepsis” (A41), the childhood and adolescence (0–19) group was the only group of patients identified in this progression (100%). No significant difference in RR was evident between men and women (females: 7.145, males: 8.182), and the median duration of progression was short (the median duration of this pair was 68 days, and the proportion of short-term progression was 88%). In contrast, in the progression from “Chronic kidney disease” (G03) to “Other sepsis” (A41), the old-age group was involved the most (81.9%). Additionally, there was a distinct RR difference between men and women (females: 10.229, males: 7.322), and progression occurred over a relatively longer time span (690.5 days).

## Discussion

Unlike the existing disease networks based on biological data or clinical data from other countries, we built a directional weighted network with explanations of the effects of predisposing factors, such as age and gender, and the progression duration information of all diagnosis pairs using claims data provided by the National Health Insurance System of Korea. We showed that our network had both in-degree and out-degree distributions following a power law, where the exponent ${\rm{\gamma }}$ is 2 < ${\rm{\gamma }}$ < 3, indicating that a few diagnoses have many out-, in- or both degrees and play a major role in the network by acting as hubs or as bridges for diagnosis progression. Furthermore, the in-degree and out-degree diagnoses were positively correlated and could be analyzed in terms of age, gender and ICD-10 categories. High in-degree and out-degree (in-degree ≥ and out-degree ≥ 4) diagnoses were relatively high in patients older than 60 years, and many low in-degree and high out-degree (in-degree < and out-degree ≥ 4) diagnoses were identified in the child and adolescent group younger than 20 years old. This result can be explained by the fact that older patients are more likely to have been exposed to various diseases and that disorders related to the perinatal period likely represent the first diagnosis in a patient’s life, explaining the low in-degree and high out-degree diagnoses. This finding is also supported by the fact that the perinatal disorders category accounted for the highest percentage of high out-degree and low in-degree diagnoses among all ICD categories. Additionally, high in- and out-degree (in-degree ≥ 4 and out-degree ≥ 4) and low in- and out-degree (in-degree < 4 and out-degree < 4) groups accounted for the highest proportions at all ages, reaffirming a strong positive correlation between in- and out-degree nodes.

Our network with high modularity suggested that nodes in the network were not randomly and evenly distributed between communities but rather clustered by properties. We showed that our network was clustered into several subgroups, with the 6 largest clusters grouped by gender, age, and ICD-10 categories as shown by the analysis of degree. Giant components, including the female-related diagnoses cluster, the component contained solely within mental illness diagnoses, and the giant cluster of diagnoses that are more common in older patients, reflect evidence that risk factors are important features in the network construction phase.

Demonstrating the relationships between diseases using only claims data is very limited. Because diagnosis errors may exist, a diagnosis itself cannot always be assumed to indicate a specific disorder^28–33^. For example, diagnosing sepsis in the early stage is very difficult because its symptoms and signs can be confused with other conditions. Therefore, it may take some time from symptom onset to confirm sepsis or the disease could be misdiagnosed initially. Since it is very difficult to conclude the exact time of disease occurrence, it is not reasonable to conclude that the nodes in the DPN directly represent diseases. Furthermore, in most cases, hospital doctors will provide various treatments such as drugs or surgery after a diagnosis is determined. Therefore, whether the next illness is the result of the previous illness or other factors is difficult to confirm. For this reason, defining edges as disease-disease relationships is problematic, which is why we introduced our network as a DPN rather than a disease network. Even though we recognize that our diagnosis progressions are not equivalent to disease-disease relationships, we assumed that the associations from our network could be used as clues to suggest differential relationships between diseases. For instance, although the two progression examples in the Results section (from “Chronic kidney disease” (G03) to “Other sepsis” (A41) and from “Disorders related to short gestation and low birth weight, NEC” (J96) to “Other sepsis” (A41)) have been studied by many researchers^34–43^, very few studies have provided or compared accurate age, gender, RR, and duration data for these two relationships. By using our network, researchers can discover new information regarding disease-disease relationships, even those that have been extensively studied in the past.

Given that many diseases have tissue specificity, the gene-disease network, which ignores tissue specificity, has many connectivities. In particular, the gene-disease network in the cancer subnetwork study deals only with the microscopic (molecular) level and does not address the macroscopic level, such as the metastasis of cancer to other organs. However, our network presented clinically meaningful connectivity and also identified connectivities that were not previously found in the gene-disease network. Compared to the gene-disease network, our network was used to verify relationship information obtained in previous research and presented new possible connectivity patterns between diseases.

Another benefit of our network is that the median duration data of all pairs are provided. The early detection of disease before symptoms or signs appear can contribute to the improvement of outcomes and survival rates for patients. If reliable information regarding the time course of disease progression to other diseases and the age groups and gender of patients at the highest risk could be obtained, the detection time could be shortened considerably and the likelihood of misdiagnosis could be decreased. Since the DPN shows the global patterns of diagnosis at a glance and suggest which diagnosis is the most feasible next step in diagnosis progression in patients with different predisposing factors, the network presented here may potentially serve as a predictive tool for the diagnosis and treatment of diseases. In addition, our goal is to build a more clinical-friendly network by constructing a network with stratification of factors such as drugs, hospitalization or treatment method. In the future, we plan to release a web service interface to the public that contains all the features of the DPN presented here and the features that will be found in future studies.

## Methods

### Source data

**Figure 6:**
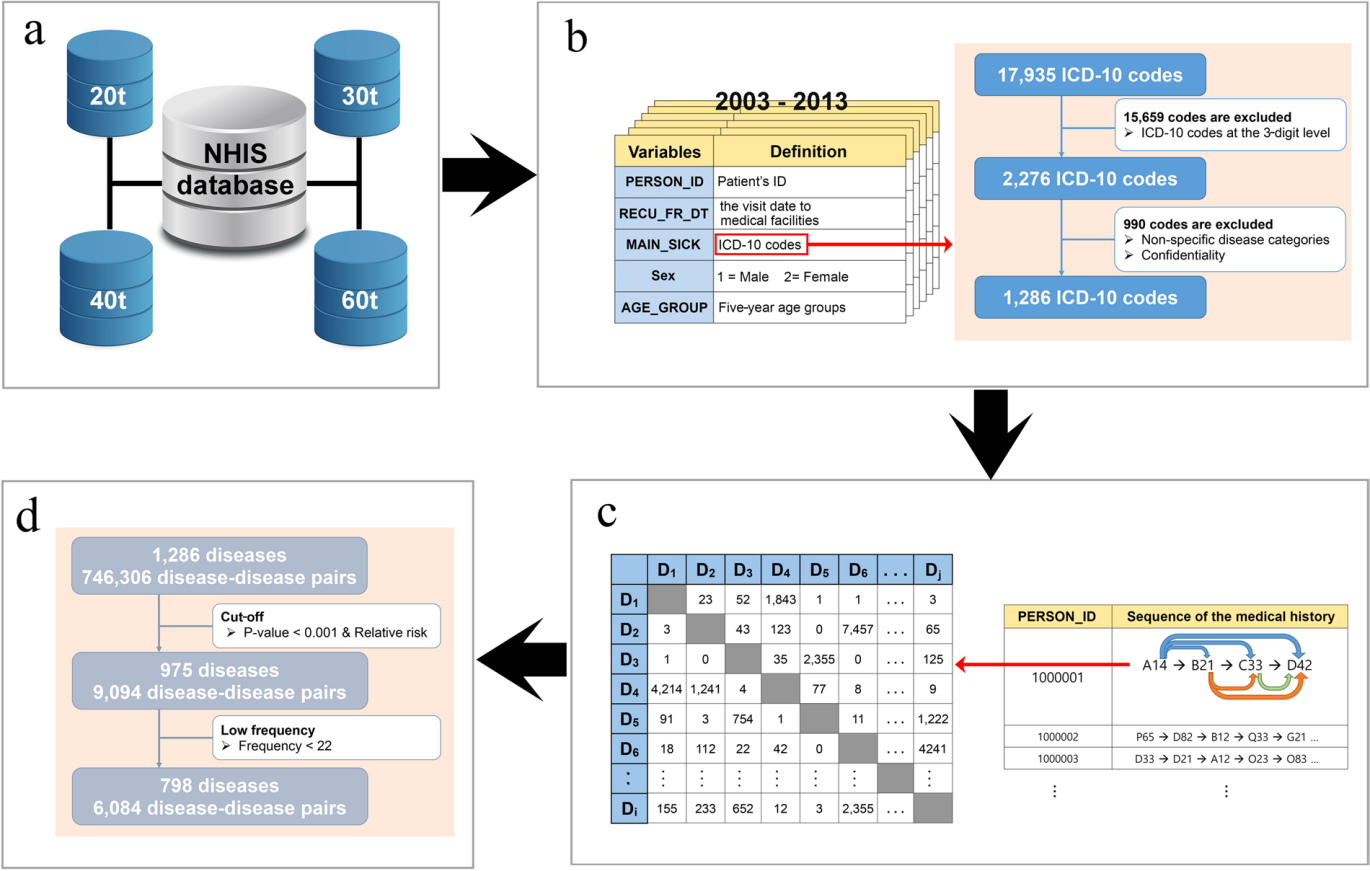
Study design and statistical analysis process. (**a**) Databases of the sample cohort data. (**b**) Five variables from the sample cohort data and the diagnosis code selection process. (**c**) Extraction of the diagnosis-diagnosis relationships and the count records in the ${D}_{i}$ x ${D}_{j}$ table. (**d**) Cutoff process to select significant pairs.

The NHIS is a universal health insurance system that covers approximately 98% of the overall Korean population. We used NHIS sample cohort data representing approximately 2% of the total Korean population from January 2002 to December 2013, covering every hospital contact including all private and public hospital visits in South Korea. The sample data consisted of the following: an eligibility database (containing patient information such as gender and age) and a medical treatment database (including electronic bills of medical treatment (20t), medical treatment bill details (30t), disease details (40t) and prescription details (60t)) (Fig. 6a). Each database contains numerous variables, of which we extracted the following: patient ID, gender, age group (in 5-year intervals), main illness (coded in the ICD-10), and visit date to medical facilities (Fig. 6b). Because of a lack of medical history data prior to 2002, the incidence data from 2002 may not be accurate. Therefore, we excluded the first-year (2002) data to avoid a detection bias regarding inaccurate diagnoses determined by incomplete data. We only considered the incidence of each diagnosis in each patient and therefore excluded cases with the same diagnosis at an earlier time. Demographically, the total population of the sample cohort data was 1,111,007 patients, with an age range of 0 to 85 + years, and 23,829,621 incidence cases were recorded.

This study was reviewed and approved by the institutional review board of the Ajou university hospital (AJIRB-MED-EXP-17-365).

### Simplification of ICD-10 codes

The Korean Classification of Diseases 6th version (KCD-6) is the Korean version of the 10^th^ revision of International Statistical Classification of Disease and Related Health Problems (ICD-10), which is the official classification for encoding diagnoses in South Korea. The only difference between the KCD-6 and the ICD-10 is that the diagnosis codes for Korean medicine are included in the KCD-6 using U20-U99 codes. We rounded all codes to 3 digits for diagnoses because the first three digits indicate the main category of the diagnosis and the last two digits provide sub category information. A 5-digit ICD-10 code consists of 17,935 disease categories, and a 3-digit ICD-10 code consists of 2,276 diseases categories. The remaining codes belonging to “Symptoms, signs and abnormal clinical and laboratory findings, NEC(R00-R99)”, “Injury, poisoning and certain other consequences of external causes (S00-T98)”, “Codes for special purposes (U00-U99)”, “External causes of morbidity and mortality (V01-Y98)”, and “Factors influencing health status and contact with health services (Z00-Z99)” were considered uncertain diagnosis terms and were excluded from the data. Under the NHIS regulation, 114 diagnosis codes were eliminated because of the privacy and security of patient health histories, and we ultimately analyzed the relationships among 1,286 ICD-10 codes (Fig. 6c).

### Extracting and filtering significant diagnosis-diagnosis associations

To identify patterns systematically, all combinations of diagnosis-diagnosis pairs from each patient’s transition were extracted using the open-source scripting language Python based on time-dependent diagnosis correlations (Fig. 6c). If a patient was diagnosed with several disorders on the same day, we assumed that those disorders were connected to each other bi-directionally. As shown in Fig. 6c, all counts of each diagnosis-diagnosis pair were recorded in the ${D}_{i}$ (the prior diagnosis) x ${D}_{j}$ (the diagnosis that appears after ${D}_{i}$) matrix, and links between diagnoses occurring in less than 22 counts, which is the same as 1 count in 50,000 patients, were eliminated (Fig. 6d). Then, the Fisher exact test with Bonferroni correction was used to reduce the risk of obtaining false-positive results. To compare the risks between diagnoses, we calculated a relative risk or risk ratio (RR), which can be expressed as follows:

1 $$R{R}_{i\to j}=\frac{{\rm{a}}\times {N}}{{\rm{b}}\times {\rm{c}}}$$

where $a$ is the counts of the *D*_*i*→*j*_ pair; *b* is the counts of pairs having *D*_*i*_ as a prior diagnosis; *c* is the counts of pairs having *D*_*j*_ as a later diagnosis; and *N* is the total counts of all diagnosis-diagnosis pairs. We regarded only those combinations with *p* < 0.001 and RR > 4 as significant. Finally, the network is represented by an $n$ x $n$ adjacency matrix, *A*_*ij*_, with elements defined as

2 $${A}_{ij}=\{\begin{array}{c}1\,{\rm{if}}\,{\rm{the}}\,{\rm{RR}}\,{\rm{of}}\,{D}_{ij}\,{\rm{pair}}\, > \,4,\\ 0\,\mathrm{otherwise},\end{array}$$

and if *A*_*ij*_ equals 1, diagnosis *i* is assumed to progress to diagnosis *j*.

The Python script is available in Supplementary Software.

### Community detection and modularity

Community structures of the network were computed using the Infomap algorithm^44–46^, which is among the best-performing network community detection methodologies^47,48^. Using techniques from data compression, Infomap detects communities by obtaining the minimum of a flow-based and information-theoretic function called the map equation, which is given as follows:

3 $$L=qH(Q)+\sum _{c=1}^{{n}_{c}}{p}_{\circlearrowright }^{c}\,qH({P}_{c})$$

where ${qH}(Q)$ represents the average number of bits required to demonstrate movement between communities, and the second term $({\sum }_{c=1}^{{n}_{c}}{p}_{\circlearrowright }^{c}qH({P}_{c}))$ indicates the average number of bits required to describe movement within communities.

Modularity, defined by Girvan and Newman, is often regarded as the quality function for community detection since it is a quantity used to measure the strength of division of a network into communities^49^. Modularity is defined as follows:

4 $${M}_{c}=\sum _{c=1}^{{n}_{c}}[\frac{{L}_{c}}{L}-{(\frac{{k}_{c}}{2L})}^{2}]$$

where *n*_*c*_ is the total number of communities; *L*_*c*_ is the total number of links within community *c*; *k*_*c*_ is the total degree of the nodes in community *c*; and $L$ is the total number of links in the full network. In general, *M*_*c*_ cannot exceed 1, and a value of *M*_*c*_ closer to 1 corresponds to better division of the network.

### Mapping from the OMIM to the ICD-10

Information regarding the mapping of OMIM terminology to ICD-9-CM codes was obtained from the supplementary table from Park *et al*.^22^. ICD-9-CM was mapped to ICD-10-CM, and then ICD-10-CM was mapped to ICD-10 using the ICD Conversion Programs implement available at https://seer.cancer.gov/tools/conversion/2017/ICD9CM_to_ICD10CM_FY2017.pdf and https://seer.cancer.gov/tools/conversion/2017/ICD10CM_to_ICD10_FY2017.pdf.

### Data availability

The sample cohort DB cannot be shared since it does not contain open data and belongs to the NHIS. Further information on the sample cohort DB can be found at https://nhiss.nhis.or.kr/bd/ab/bdaba022eng.do.

## Electronic supplementary material

Supplementary Table S1

Supplementary Table S2

Supplementary Table S3

Supplementray Software
